# Bayesian identification of bacterial strains from sequencing data

**DOI:** 10.1099/mgen.0.000075

**Published:** 2016-08-25

**Authors:** Aravind Sankar, Brandon Malone, Sion C. Bayliss, Ben Pascoe, Guillaume Méric, Matthew D. Hitchings, Samuel K. Sheppard, Edward J. Feil, Jukka Corander, Antti Honkela

**Affiliations:** ^1^​ Helsinki Institute for Information Technology, Department of Computer Science, University of Helsinki, Helsinki, Finland; ^2^​ German Centre for Cardiovascular Research DZHK, Klaus Tschira Institute for Integrative Computational Cardiology and Department of Internal Medicine III, University of Heidelberg, Germany; ^3^​ Department of Biology and Biochemistry, University of Bath, UK; ^4^​ Institute of Life Sciences, College of Medicine, Swansea University, UK; ^5^​ Helsinki Institute for Information Technology, Department of Mathematics and Statistics, University of Helsinki, Helsinki, Finland; ^6^​ Department of Biostatistics, University of Oslo, Norway

**Keywords:** pathogenic bacteria, strain identification, staphylococcus aureus, probabilistic modelling

## Abstract

Rapidly assaying the diversity of a bacterial species present in a sample obtained from a hospital patient or an environmental source has become possible after recent technological advances in DNA sequencing. For several applications it is important to accurately identify the presence and estimate relative abundances of the target organisms from short sequence reads obtained from a sample. This task is particularly challenging when the set of interest includes very closely related organisms, such as different strains of pathogenic bacteria, which can vary considerably in terms of virulence, resistance and spread. Using advanced Bayesian statistical modelling and computation techniques we introduce a novel pipeline for bacterial identification that is shown to outperform the currently leading pipeline for this purpose. Our approach enables fast and accurate sequence-based identification of bacterial strains while using only modest computational resources. Hence it provides a useful tool for a wide spectrum of applications, including rapid clinical diagnostics to distinguish among closely related strains causing nosocomial infections. The software implementation is available at https://github.com/PROBIC/BIB.

## Data Summary

1. Benchmarking data have been deposited in Figshare; DOI: 10.6084/m9.figshare.1617539 (url – http://figshare.com/articles/Benchmarking_data_for_bacterial_strain_identification/1617539)

## Impact Statement

Bacterial samples can today be routinely scrutinized with in-depth sequencing covering the majority of the genomes of the organisms present. This provides a basis for discovering the presence of mixed colonies and identification of the relative abundances of different strains at subspecies level. Here we present a novel analysis pipeline by which such identification problems can be solved considerably more rapidly and accurately compared with the existing methods, while only using modest computational resources. Our pipeline is a useful tool for a wide spectrum of applications, including contamination detection and rapid clinical diagnostics to distinguish among closely related strains causing nosocomial infections.

## Introduction

Different strains of pathogenic bacteria are known to often vary in terms of virulence, resistance and geographical spread (Méric *et al.*, 2015). Rapid and inexpensive sequence-based identification of the strain(s) colonising a patient would be highly desirable. Previous research shows that patients can often host several strains of specific species of the genus *Staphylococcus* (Ueta *et al.*, 2007). The current approach is to isolate single colonies, assuming that the sample is homogeneous. Here we consider an approach which allows a robust test of this assumption, and if there is diversity, an efficient means to compare similarities between whole host populations present in different individuals. Since single random colonies might be misleading, it is beneficial to allow for a more flexible approach where pooled colony data can be directly utilized.

With the growing tendency to routinely sequence samples from infected patients in the hospital environment, the identification would be additionally advantageous for pathogen surveillance and monitoring purposes without necessitating the use of extensive computational resources for *de novo *genome assembly. Moreover, samples with mixed presence of several strains are problematic for assembly-based analyses, which calls for alternative approaches.

The identification of bacteria from sequencing data has been widely considered in metagenomic community profiling (Segata *et al.*, 2013; Franzosa *et al.*, 2015). As our primary identification and estimation focus is at a much higher level of resolution than in typical metagenomics studies, whole-genome or whole-metagenome shotgun sequencing data is by definition a necessity for a successful implementation of a platform for this purpose. Typical metagenomic approaches for such data are based on defining a set of markers for each clade of interest (Segata *et al.*, 2012; Sunagawa *et al.*, 2013). However, these methods are typically not sensitive enough to identify the pathogens responsible for infections in sufficient detail. Eyre *et al.* (2013) have presented a method for detecting mixed infections but the method assumes there are at most two strains in each sample, which may not hold, in particular if a sample has become contaminated at any phase of the preparation and sequencing process. A Bayesian statistical method capable of using all the sequencing data was recently introduced (Francis *et al.*, 2013; Hong *et al.*, 2014), but also its practical performance may not be appropriate, as suggested by our experiments.

The computational problem in bacterial strain identification is analogous to the widely studied transcript isoform expression estimation in RNA-sequencing (RNA-seq) data analysis, which both aim at identifying and quantifying the abundance of several closely related sequences from short-read data. In both cases a significant fraction of reads will align perfectly to multiple sequences of interest. Several probabilistic models have been proposed for solving this problem (Xing *et al.*, 2006; Jiang & Wong, 2009). Based on its success in recent assessments of methods for tackling this problem (SEQC/MAQC-III Consortium, 2014; Kanitz *et al.*, 2015), we use the BitSeq (Glaus *et al.*, 2012; Hensman *et al.*, 2015) method to obtain a fast and accurate solution to this problem in our Bayesian Identification of Bacteria (BIB) pipeline for bacterial strain identification from unassembled sequence reads.

In this paper we focus on *Staphylococcus aureus *and *Staphylococcus epidermidis*, which represent two of the most widespread causes of nosocomial infections and impose considerable burden on the public health system worldwide (Harris *et al.*, 2010; Méric *et al.*, 2015). Using a diverse collection of strains from these two species as a model system, we demonstrate that clinically relevant, fast and highly accurate identification of the strains colonising a patient is possible in less than 10 min on a standard single-CPU desktop computer. Our BIB pipeline improves significantly upon the state-of-the-art approach for sequence-based identification of bacteria.

## Methods

Our pipeline is built by a combination of the following two central ideas:

1. defining core genomes of the target set of strains by excluding more variable regions to strengthen the analysis, and
2. using a fast fully probabilistic method to estimate the relative frequencies of the target strains in a sample.

These ideas translate to two analysis steps:

Step 1. Cluster the strains, perform multiple sequence alignment to find the strain-specific common core genome and construct an index for read alignment.

Step 2. Align the reads to the reference core genomes allowing multiple matches and use a probabilistic method to estimate the strain abundances using the alignments.

Step 1 only needs to be done once for each collection of reference sequences while Step 2 needs to be performed for every sample. The two steps will be detailed further below, followed by description of the synthetic data generation process and characterisation of the real data which are used for empirical evaluation and comparison against the leading alternative identification method.

### Step 1: Reference strain selection and core genome extraction.

**Fig. 1.**
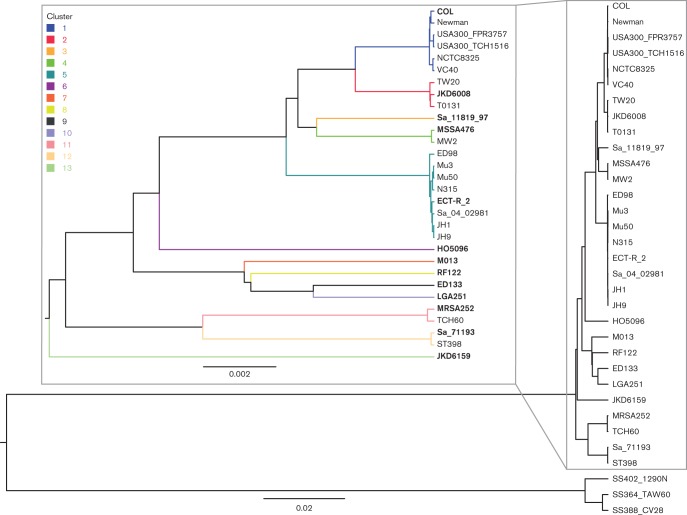
Phylogenetic tree of the investigated *Staphylococcus* strains. Inset: Enlarged view of the *S. aureus* branch illustrating the clustering of the strains within clonal complexes. The scale measures base-level sequence dissimilarity, showing that the *S. aureus* clusters differ by approximately two to ten substitutions every 1 kb while strains within each cluster differ by less than one substitution every 5 kb.

We demonstrate our pipeline on a collection of 30 *S. aureus *and 3 *S. epidermidis *strains whose phylogenetic tree is illustrated in Fig. 1. The tree was reconstructed using UPGMA method with p-distance in the mega6 software (Tamura *et al.*, 2013). The tree displays a natural partition with 13 *S. aureus *strain clusters, each of which corresponds to an already established clonal complex (Feil & Enright, 2004), while each *S. epidermidis *strain forms a cluster of its own, representing the three previously identified main complexes within the species (Méric *et al.*, 2015). The strains selected to represent each cluster are indicated by bold type in Fig. 1.

Microbial genomes are often highly dynamic and susceptible to horizontal gene transfer and translocation of genomic regions (Gogarten *et al.*, 2002; Lawrence, 2002). As a consequence, mobile elements may confuse genome-based identification of strains. In order to avoid issues with misalignment of reads and incorrect abundance estimates, we discard the non-core parts of the reference genomes and use only core alignment, i.e. parts of the genome shared by all strains of a species, as a basis for the analysis.

A multiple sequence alignment for the 16 cluster prototype bacterial strains shown in bold in Fig. 1 was obtained using progressive Mauve (Darling *et al.*, 2010). The accessory genome regions were detected and discarded using the standard settings, resulting in an ungapped core alignment which was used to represent the genomic variation in the target set of strains. These ungapped sequences are used to construct an index for read alignment.

### Step 2: Strain abundance estimation.

The gapless core genomes extracted as described above were considered as the reference sequences in the BitSeq (Glaus *et al.*, 2012; Hensman *et al.*, 2015) method to estimate the relative proportion of each strain in our reference collection in a sample. We used Bowtie 2 (Langmead & Salzberg, 2012) to align the reads to the reference sequences allowing for multiple matches. We then used estimateVBExpression from BitSeq to estimate the relative proportions of each of the strains in the sequenced samples. Our full method pipeline is referred to as Bayesian Identification of Bacteria (BIB) in the remainder of the article.

### Abundance estimation model in detail.

The strain abundance estimation was based on a statistical model of sequencing data as a mixture of reads from a set of known reference sequences (Xing *et al.*, 2006; Li *et al.*, 2010). The relative abundances of the sequences are the unknown parameters θ. In our case the references were the core genomes of randomly selected representatives of each cluster. Reads not mapping to the core genomes were ignored.

After introducing indicator variables *I_n_* defining the sequence of origin of each read *r_n_*, the likelihood of a read *r_n_* (single or paired-end) *p(r_n_|I_n_ = m)* is defined in Equation (1) of Glaus *et al.* (2012) and depends on the mismatches in the alignment as well as the length of the reference sequence. The position model was not used in BIB because it would be difficult to estimate with almost no unique alignments. We used a conjugate Dirichlet(α,...,α) prior over θ with α = 1. Smaller α would mean weaker regularisation, but α ≥ 1 is needed for log-concavity of the model, which aids convergence.

We used fast collapsed variational inference to optimise an approximate posterior distribution over *I_n_* after marginalising out θ (Hensman *et al.*, 2012, 2015). The posterior distribution over the unknown abundances θ was obtained from these as in Hensman *et al.* (2015).

### Generation of data for validation experiments.

For the primary set of experiments, each sample was created by randomly mixing the reads from a number of real single-strain sequencing data sets (Data citations 1–3). Details of the strains and the used mixing proportions are provided in Table S1 (available in the online Supplementary Material) and the full data set is available through Data citation 4. These data are obtained independently of the reference sequences used in the model and represent realistic sequencing data obtained from other strains in the same clusters.

To test more thoroughly the effect of dropped clusters in the presence of a more diverse representation of different strains, we additionally simulated reads using MetaSim (Richter *et al.*, 2008).

## Results

We tested the BIB pipeline on several DNA sequencing data sets from *Staphylococcus *strains. We used two different types of data sets:

1. data sets with artificial mixtures of genuine reads from single strain sequencing experiments, and
2. synthetic data sets generated using MetaSim.

We report the results from our pipeline and compare against Pathoscope 2 (Francis *et al.*, 2013; Hong *et al.*, 2014) as well as naive estimation from strain frequencies among uniquely mapping reads. To ensure that the other methods can fully utilise the same information about the strains, we used the same read alignments as input to all methods, essentially only replacing the final abundance estimation step in our pipeline.

### Clustering and selection of strains

The strains used in the experiment and their phylogenetic relationships are illustrated in Fig. 1. The phylogenetic tree illustrates the clonal complex (CC) structure of the *S. aureus *population (Feil & Enright, 2004), where members of the same complex are highly similar and interchangeable in terms of strain identification (Méric *et al.*, 2015). Choosing one representative for each CC corresponds to the clustering illustrated in Fig. 1.

### Identification of *Staphylococcus *strains from sequencing data

We generated 30 synthetic mixtures of sequencing reads from different strains of species of the genus *Staphylococcus *as described and analysed these data sets using BIB. As a benchmark, we also tested the same identification and quantification using Pathoscope instead of BitSeq. Each analysed data set contained a mixture of two to six *Staphylococcus *strains. The number of reads varied between one million and three million. The data sets mixed from previous data (Data citations 1–3) are available in Figshare (Data citation 4). Details of the samples and mixing proportions are given in Table S1.

Strain-level identification is very difficult, as typically only around 0.1–0.2 % of the reads map uniquely to the core genome. Full genome alignments have more unique hits, but given the volatility of the accessory genome these are also likely to be more misleading.

**Fig. 2.**
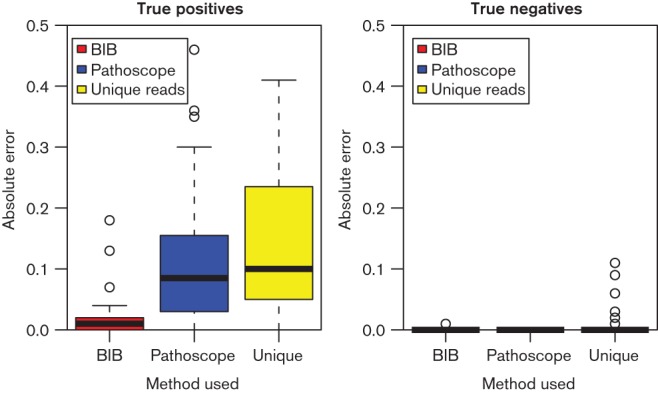
Magnitudes of errors in proportion estimates of BIB, Pathoscope and naive estimation among uniquely mapping reads (Unique) in strains really present in the experiment (true positives; left) and those not present in the experiment (true negatives; right). The “Unique” method is implemented by simply computing the frequencies of different strain clusters among unique alignments. Lower values indicate better results.

**Fig. 3.**
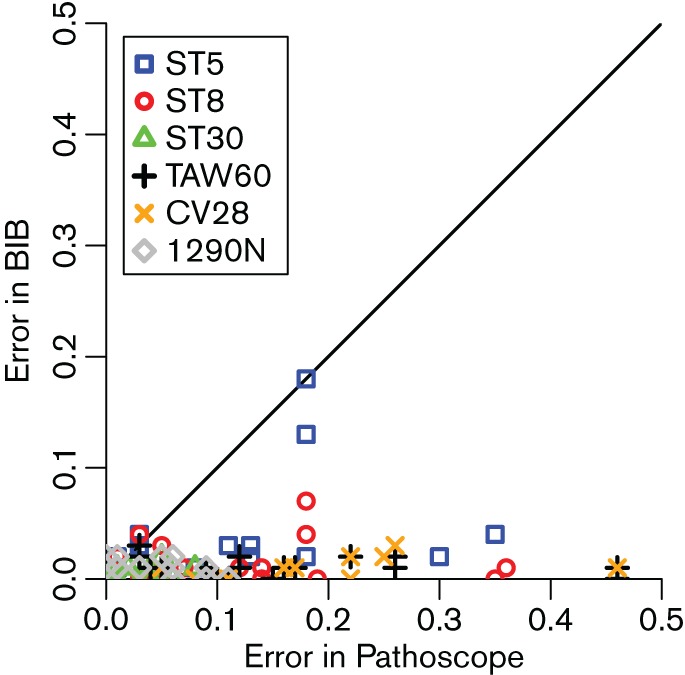
Scatter plot comparing the estimation errors of BIB and Pathoscope on true positives. Points below the diagonal are cases where BIB is more accurate while points above the diagonal are cases where Pathoscope is more accurate.

The absolute errors in the abundance estimation in the experiments are illustrated in Fig. 2. We split our analysis to two cases: strains not present in the samples (true negatives) and strains that are present (true positives). All methods are reliable in identifying true negatives. For true positives, BIB consistently provides very accurate quantification (absolute errors mean ± sd 0.014 ± 0.023) while Pathoscope and the naive unique mapping read analysis are significantly less accurate (Pathoscope absolute errors 0.11 ± 0.11, unique reads 0.14 ± 0.12). BIB quantification results remain accurate all the way down to the least abundant strains which had only 3 % abundance in our data. A scatter-plot in Fig. 3 comparing the errors of BitSeq and Pathoscope for each experiment shows that BIB is essentially always more accurate than Pathoscope (*P < *10^−16^; Wilcoxon signed rank test) and often by a wide margin.

### Estimation in the presence of contaminant species

**Fig. 4.**
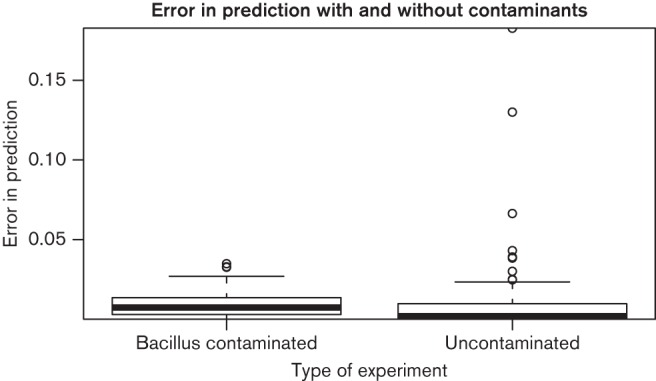
Comparison of errors in estimation of proportions of *Staphylococcus *strains with and without *Bacillus* contamination. Errors on contaminated samples are slightly higher, but overall still very low.

The alignment of reads to reference genomes makes BIB highly robust to contamination by unrelated species. We tested this by generating ten of the samples with 3–30 % contamination from *Bacillus subtilis* subsp.* subtilis *strain 168. Full details of the experiments are given in Table S1. After filtering the non-aligning reads, which include most of the *Bacillus *reads, the estimation accuracy on the proportions of *Staphylococcus* strains is almost as good as with the uncontaminated samples, as illustrated in Fig. 4. The corresponding median errors are 0.002 for the uncontaminated samples and 0.01 for the contaminated samples, respectively. Addition of the contaminant reads is visible as a drop in the total rate of aligned reads, but given the significant and variable number of unmappable reads originating from the auxiliary genome the mapping rate is at best an unreliable measure of the contamination level.

### Estimation in the presence of unknown strain clusters

**Fig. 5.**
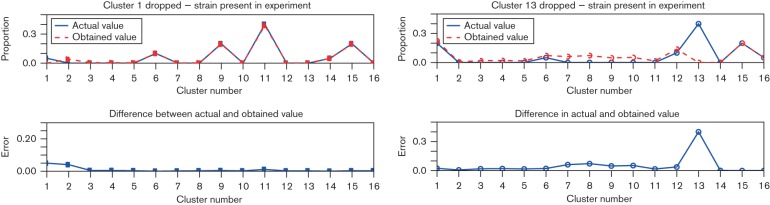
Two examples of error spectra when some strain clusters present in a sample are not included in the index. The plots show the profile of true and estimated proportions (top) as well as the errors in the estimation (bottom). The error profile lines will always show a bump at the dropped cluster index because they cannot be estimated while the other shape shows how the reads get reassigned.

When the reads of unknown origin stem from a species or strain related closely enough to allow for the reads aligning well with those included in the index, they tend to be assigned to the most closely related included reference strains. This is illustrated by two examples in Fig. 5. In the first example dropping Cluster 1 from the index causes the reads to get assigned to Cluster 2 which is in the evolutionary sense closest to Cluster 1 in the phylogenetic tree in Fig. 1. In the second example, dropping Cluster 13, results in the reads getting split more evenly among the available alternatives because the branch to Cluster 13 splits off from the rest very early.

### Estimation without clustering

**Fig. 6.**
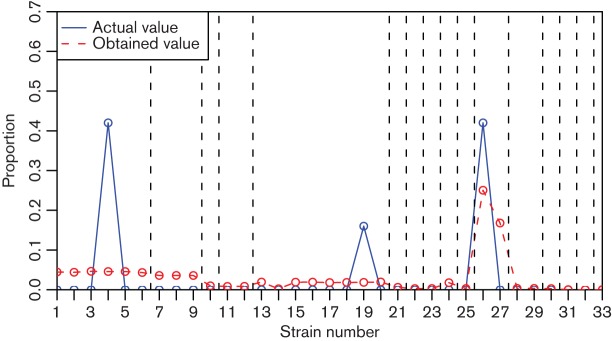
An example of error profile in strain abundance estimation without clustering. The vertical dotted lines indicate the borders between different clusters.

Clustering of very similar strains when defining the reference set is an essential part of BIB. Fig. 6 shows a typical example of the consequences of excluding the clustering step. As seen, the contribution of a single cluster representative truly present in a sample tends to get split up between all strains representing the same cluster in the reference set as they are too similar to be differentiated. Furthermore, the method is unable to separate strains 1–9 belonging to Clusters 1 and 2, even though the two were usually properly separated in the experiments with clustering of the reference strains. This is most likely because the difference from using six or nine strains to represent the data is not as substantial as the difference between one or two strain clusters where the clearly simpler model is able to drive the other coefficient to zero. It is likely that no statistical method would be able to truthfully resolve the origins of the reads when the sources are too similar to each other. Hence, it is of importance to ensure biological meaningfulness of the reference set of strains prior to assignment.

### Analysis of clinical *S. aureus* samples

**Fig. 7.**
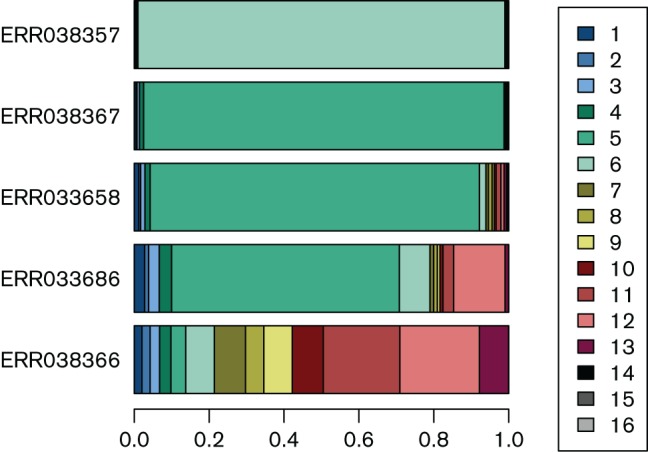
Estimated cluster abundance profiles from diverse clinical samples. The two top rows represent clean samples where one cluster clearly dominates. Rows 3 and 4 represent contaminated samples where the true cluster can still be fairly reliably identified. The bottom row shows a completely failed sample, possibly due to problems with sequence barcoding.

To illustrate the practical applicability of BIB we tested it on *S. aureus* short-read data generated at the Wellcome Trust Sanger Institute as part of a Europe-wide surveillance project [“Genetic diversity in *Staphylococcus aureus* (European collection)” study; Data citation 5], with kind permission from Matthew Holden. Initial analysis of these data revealed they were of poor quality, probably resulting from contamination, and for this reason they have not previously been published. All isolates were recovered from cases of invasive *S. aureus* disease. The estimated abundance profiles of selected samples are shown in Fig. 7. In top two isolates (ERR038357 and ERR038367) a single cluster is robustly identified (> 95 % share for the dominant strain, all other shares < 1 %) indicating that the level of contamination in these samples is low. In contrast, isolates ERR033658 and ERR033686 (rows 3 and 4) show clearer evidence of mixed clusters due to contamination. We also note that the cluster profiles are similar within these two samples, which is consistent with a single source of contamination for both runs. Isolate ERR038366 (bottom row) represents a completely failed sample, possibly caused by problems with sequence barcoding.

### Analysis of samples of a more recombinant species *S. epidermidis*

**Fig. 8.**
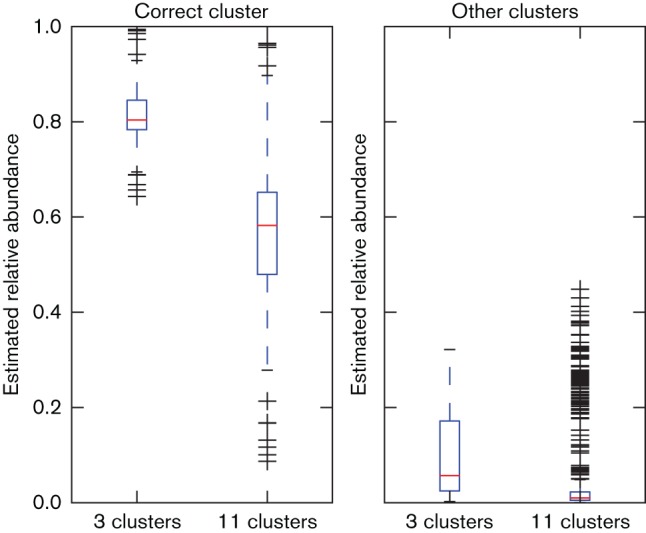
Estimated relative abundances of the correct and other clusters in *S. epidermidis* data with two different clusterings. (Higher values are better for correct cluster, lower values for other clusters.) The results show more leakage of estimates to incorrect clusters, but the true cluster is still identified as dominant in all cases with three clusters and in 85 % of cases with 11 clusters.

To test the applicability of BIB to more recombinant species than *S. aureus*, we applied it to 83 *S. epidermidis* isolates sequenced by Méric *et al.* (2015) (Data citation 6, details inTable S2). We analysed the samples using the two clusterings of an extended set of 143 strains analysed by Méric *et al.* (2015): a coarse clustering representing a clinically relevant partition of the population into three clonal complexes, and a more detailed clustering into 11 groups which subdivides the clonal complexes further. We randomly selected representatives from each cluster as prototype isolates in the BIB database. Samples used as prototypes were excluded from the analysis, leaving 82 samples in the three-cluster case and 75 in the 11-cluster case. The estimated relative abundances of the true and other clusters are shown in Fig. 8. The figure shows that in the case of the clinically motivated population partition the correct cluster is always identified as dominant with a large margin, but the absolute errors are larger than in the *S. aureus* case, typically around 20 %. The results in the 11-cluster case show more variability, but the correct cluster is still identified as dominant in 85 % of the cases and the abundance estimates for the other clusters are in most cases very small.

### Runtime

For a new sample, the pipeline requires running of programs for read alignment (Bowtie 2) and abundance estimation (BitSeq being the core part of BIB). The time required by these two steps is approximately equal. A typical analysis of 1 M reads takes approximately 10 min on a single-CPU desktop computer representing a standard level of hardware.

## Discussion

### Interpretation of proportions and benchmarking

Our pipeline can estimate strain abundances as proportions of the sequencing reads. These would be expected to be related to the proportions of DNA from the different strains. Depending on the relative lengths of different genomes, this may deviate slightly from cell counts between species, but should be consistent within a species because we only consider the shared core genome of equal length. This kind of minor variations should not affect any practical applications.

Our empirical evaluation is based solely on synthetic mixtures of sequencing reads from different single-strain sequencing experiments. Such mixtures are necessary to enable accurate benchmarking of the methods. Because we use actual reads from various experiments they will not perfectly match the reference and thus represent a much more realistic test than synthetic reads generated from references. Experiments based on laboratory-derived mixed cultures would add significant extra uncertainty because it is difficult to accurately control the strain proportions during the cultivation process.

### Applicability to different bacterial species

The main assumption behind our BIB method is that each putative biologically meaningful source is adequately represented by a single core genome sequence to which the reads can be mapped. As illustrated in this paper, this works with high fidelity for species like *S. aureus *whose population structure has clear well-separated lineages (Méric *et al.*, 2015). The results with more highly recombining species such as *S. epidermidis* show more variability in the proportion estimates, but the correct cluster is still always identified as the dominant one in the clinically most relevant three-cluster case and also in a great majority of samples in the more detailed scenario as well. Improving the performance for more recombinant species is an important avenue of future work. The current state-of-the-art probabilistic identification method Pathoscope 2 (Francis *et al.*, 2013; Hong *et al.*, 2014) is essentially based on a similar assumption and is expected to be similarly vulnerable to strong deviations from the assumption. However, our experiments demonstrated that BIB delivers a considerably higher level of estimation accuracy without requiring more extensive computational resources.

As illustrated in the results shown in Fig. 6, clustering the strains is essential for accurate identification results. It is not surprising that distinguishing among multiple highly related strains is not feasible, however, it is more striking that clustering also aids identification of read origin between the more separated sources. We suspect this may be due to the prior used in the Bayesian model, but further work is needed to properly understand the phenomenon.

In transcript-level RNA-seq analysis clustering of similar transcripts has been suggested for improving the accuracy by Turro *et al.* (2014). Unlike our off-line clustering, their algorithm is run on-line together with the inference separately for every sample. Our approach can easily incorporate additional expert knowledge and guarantee consistent clustering, making interpretation of the results more straightforward. This approach is expected to work especially well for any species that has a clear subpopulation boundaries, since every potentially mixed sample will correspondingly have a clearly delineable structure among its reads, apart from those representing contamination, which can be efficiently filtered out by our pipeline.

The *S. epidermidis* results highlight a trade-off in the number of clusters: increasing the number of clusters raises the risk of misidentification while providing potentially more relevant information. The optimal number of clusters clearly depends on the aim of the analysis.

Selection of the representative sequence for each cluster for more highly recombinant species like *S. epidermidis* is an interesting question for future work. It seems that the optimal answer will depend on the assumed distribution of analysed samples. Presumably one could simulate data from the non-selected representatives in each cluster and test the method, but this could become quite computationally demanding, as the choices in different clusters are clearly not independent. Here we have used random selection to avoid such questions.

### Relationship to transcript-level RNA-seq analysis

The underlying statistical problem in bacterial strain identification is the same as that underlying most transcript-level RNA-seq expression estimation methods: how to estimate the probability of a read originating from a given reference sequence. There exist a number of methods solving the same problem there including RSEM (Li *et al.*, 2010; Li & Dewey, 2011), Cufflinks (Trapnell *et al.*, 2010), Miso (Katz *et al.*, 2010), BitSeq (Glaus *et al.*, 2012; Hensman *et al.*, 2015), TIGAR (Nariai *et al.*, 2013, 2014), eXpress (Roberts & Pachter, 2013), Sailfish (Patro *et al.*, 2014) and many others. These are all based on different inference methods applied to the same probabilistic model first proposed in (Xing *et al.*, 2006). This is also essentially the same as the model used by Pathoscope (Francis *et al.*, 2013; Hong *et al.*, 2014). There are also a number of other RNA-seq analysis methods based on other models. We have chosen to use the fast variational Bayes (VB) version of BitSeq (Hensman *et al.*, 2015) as core ingredient in BIB because it provides very high accuracy while being reasonably fast according to recent broad assessments (SEQC/MAQC-III Consortium, 2014; Kanitz *et al.*, 2015).

## Conclusion

In this paper we have presented the BIB pipeline for probabilistic identification and quantification of relative abundance of bacterial strains in mixed samples from unassembled sequence data. The pipeline is based on alignment of reads to representative core genomes followed by deconvolution of multi-mapping reads using BitSeq, a method previously proposed for RNA-seq analysis. Our BIB pipeline can rapidly and reliably estimate the proportions of the reference strains with the typical deviance of at most a few percent units, using approximately one million sequencing reads. BIB improves significantly upon the accuracy of both naive analysis as well as previous state-of-the-art method in strain identification. Application of BIB to analyse clinical samples suggests it has significant potential both in strain identification as well as flagging problematic, such as contaminated, samples.

## Supplementary Data

38 Supplementary File 1

## Supplementary Data

71 Supplementary File 2

## References

[R1] Darling A. E. Mau B. Perna N. T.(2010). progressiveMauve: multiple genome alignment with gene gain, loss and rearrangement. PLoS One 5 e11147. 10.1371/journal.pone.0011147 20593022 PMC2892488

[R2] Eyre D. W. Cule M. L. Griffiths D. Crook D. W. Peto T. E. Walker A. S. Wilson D. J.(2013). Detection of mixed infection from bacterial whole genome sequence data allows assessment of its role in *Clostridium difficile* transmission. PLoS Comput Biol 9 e1003059. 10.1371/journal.pcbi.1003059 23658511 PMC3642043

[R3] Feil E. J. Enright M. C.(2004). Analyses of clonality and the evolution of bacterial pathogens. Curr Opin Microbiol 7 308–313. 10.1016/j.mib.2004.04.002 15196500

[R4] Francis O. E. Bendall M. Manimaran S. Hong C. Clement N. L. Castro-Nallar E. Snell Q. Schaalje G. B. Clement M. J.(2013). Pathoscope: species identification and strain attribution with unassembled sequencing data. Genome Res 23 1721–1729. 10.1101/gr.150151.112 23843222 PMC3787268

[R5] Franzosa E. A. Hsu T. Sirota-Madi A. Shafquat A. Abu-Ali G. Morgan X. C. Huttenhower C.(2015). Sequencing and beyond: integrating molecular 'omics' for microbial community profiling. Nat Rev Microbiol 13 360–372. 10.1038/nrmicro3451 25915636 PMC4800835

[R6] Glaus P. Honkela A. Rattray M.(2012). Identifying differentially expressed transcripts from RNA-seq data with biological variation. Bioinformatics 28 1721–1728. 10.1093/bioinformatics/bts260 22563066 PMC3381971

[R7] Gogarten J. P. Doolittle W. F. Lawrence J. G.(2002). Prokaryotic evolution in light of gene transfer. Mol Biol Evol 19 2226–2238. 10.1093/oxfordjournals.molbev.a004046 12446813

[R8] Harris S. R. Feil E. J. Holden M. T. Quail M. A. Nickerson E. K. Chantratita N. Gardete S. Tavares A. Day N.(2010). Evolution of MRSA during hospital transmission and intercontinental spread. Science 327 469–474. 10.1126/science.1182395 20093474 PMC2821690

[R9] Hensman J. Rattray M. Lawrence N. D.(2012). Fast variational inference in the conjugate exponential family. Advances in Neural Information Processing Systems 25 2888–2896. Edited by Pereira F. Burges C. J. C. Bottou L. Weinberger K. Q. La Jolla, CA, USA: Neural Information Processing Systems Foundation.

[R10] Hensman J. Papastamoulis P. Glaus P. Honkela A. Rattray M.(2015). Fast and accurate approximate inference of transcript expression from RNA-seq data. Bioinformatics 31 3881–3889. 10.1093/bioinformatics/btv483 26315907 PMC4673974

[R11] Hong C. Manimaran S. Shen Y. Perez-Rogers J. F. Byrd A. L. Castro-Nallar E. Crandall K. A. Johnson W. E.(2014). PathoScope 2.0: a complete computational framework for strain identification in environmental or clinical sequencing samples. Microbiome 2 33. 10.1186/2049-2618-2-33 25225611 PMC4164323

[R12] Jiang H. Wong W. H.(2009). Statistical inferences for isoform expression in RNA-Seq. Bioinformatics 25 1026–1032. 10.1093/bioinformatics/btp113 19244387 PMC2666817

[R13] Kanitz A. Gypas F. Gruber A. J. Gruber A. R. Martin G. Zavolan M.(2015). Comparative assessment of methods for the computational inference of transcript isoform abundance from RNA-seq data. Genome Biol 16 150. 10.1186/s13059-015-0702-5 26201343 PMC4511015

[R14] Katz Y. Wang E. T. Airoldi E. M. Burge C. B.(2010). Analysis and design of RNA sequencing experiments for identifying isoform regulation. Nat Methods 7 1009–1015. 10.1038/nmeth.1528 21057496 PMC3037023

[R15] Langmead B. Salzberg S. L.(2012). Fast gapped-read alignment with Bowtie 2. Nat Methods 9 357–359. 10.1038/nmeth.1923 22388286 PMC3322381

[R16] Lawrence J. G.(2002). Gene transfer in bacteria: speciation without species? Theor Popul Biol 61 449–460. 10.1006/tpbi.2002.1587 12167364

[R17] Li B. Ruotti V. Stewart R. M. Thomson J. A. Dewey C. N.(2010). RNA-Seq gene expression estimation with read mapping uncertainty. Bioinformatics 26 493–500. 10.1093/bioinformatics/btp692 20022975 PMC2820677

[R18] Li B. Dewey C. N.(2011). RSEM: accurate transcript quantification from RNA-Seq data with or without a reference genome. BMC Bioinformatics 12 323. 10.1186/1471-2105-12-323 21816040 PMC3163565

[R19] Méric G. Miragaia M. De Been M. Yahara K. Pascoe B. Mageiros L. Mikhail J. Harris L. G. Wilkinson T. S.(2015). Ecological overlap and horizontal gene transfer in *Staphylococcus aureus* and *Staphylococcus epidermidis*. Genome Biol Evol 7 1313–1328. 10.1093/gbe/evv066 25888688 PMC4453061

[R20] Nariai N. Hirose O. Kojima K. Nagasaki M.(2013). TIGAR: transcript isoform abundance estimation method with gapped alignment of RNA-Seq data by variational Bayesian inference. Bioinformatics 29 2292–2299. 10.1093/bioinformatics/btt381 23821651

[R21] Nariai N. Kojima K. Mimori T. Sato Y. Kawai Y. Yamaguchi-Kabata Y. Nagasaki M.(2014). TIGAR2: sensitive and accurate estimation of transcript isoform expression with longer RNA-Seq reads. BMC Genomics 15 S5. 10.1186/1471-2164-15-S10-S5 25560536 PMC4304212

[R22] Patro R. Mount S. M. Kingsford C.(2014). Sailfish enables alignment-free isoform quantification from RNA-seq reads using lightweight algorithms. Nat Biotechnol 32 462–464. 10.1038/nbt.2862 24752080 PMC4077321

[R23] Richter D. C. Ott F. Auch A. F. Schmid R. Huson D. H.(2008). MetaSim: a sequencing simulator for genomics and metagenomics. PLoS One 3 e3373. 10.1371/journal.pone.0003373 18841204 PMC2556396

[R24] Roberts A. Pachter L.(2013). Streaming fragment assignment for real-time analysis of sequencing experiments. Nat Methods 10 71–73. 10.1038/nmeth.2251 23160280 PMC3880119

[R25] SEQC/MAQC-III Consortium(2014). A comprehensive assessment of RNA-seq accuracy, reproducibility and information content by the sequencing quality control consortium. Nat Biotechnol 9 903–914. 10.1038/nbt.2957 PMC4321899 25150838

[R26] Segata N. Waldron L. Ballarini A. Narasimhan V. Jousson O. Huttenhower C.(2012). Metagenomic microbial community profiling using unique clade-specific marker genes. Nat Methods 9 811–814. 10.1038/nmeth.2066 22688413 PMC3443552

[R27] Segata N. Boernigen D. Tickle T. L. Morgan X. C. Garrett W. S. Huttenhower C.(2013). Computational meta'omics for microbial community studies. Mol Syst Biol 9 666. 10.1038/msb.2013.22 23670539 PMC4039370

[R28] Shiwa Y. Matsumoto T. Yoshikawa H.(2013). Identification of laboratory-specific variations of* Bacillus subtilis *strains used in Japan. Biosci Biotechnol Biochem 77 2073–2076. 10.1271/bbb.130438 24096670

[R29] Sunagawa S. Mende D. R. Zeller G. Izquierdo-Carrasco F. Berger S. A. Kultima J. R. Coelho L. P. Arumugam M. Tap J.(2013). Metagenomic species profiling using universal phylogenetic marker genes. Nat Methods 10 1196–1199. 10.1038/nmeth.2693 24141494

[R30] Tamura K. Stecher G. Peterson D. Filipski A. Kumar S.(2013). mega6: molecular evolutionary genetics analysis version 6.0. Mol Biol Evol 30 2725–2729. 10.1093/molbev/mst197 24132122 PMC3840312

[R31] Trapnell C. Williams B. A. Pertea G. Mortazavi A. Kwan G. Van Baren M. J. Salzberg S. L. Wold B. J. Pachter L.(2010). Transcript assembly and quantification by RNA-Seq reveals unannotated transcripts and isoform switching during cell differentiation. Nat Biotechnol 28 516–520. 10.1038/nbt.1621 20436464 PMC3146043

[R32] Turro E. Astle W. J. Tavaré S.(2014). Flexible analysis of RNA-seq data using mixed effects models. Bioinformatics 30 180–188. 10.1093/bioinformatics/btt624 24281695

[R33] Ueta M. Iida T. Sakamoto M. Sotozono C. Takahashi J. Kojima K. Okada K. Chen X. Kinoshita S. Honda T.(2007). Polyclonality of *Staphylococcus epidermidis* residing on the healthy ocular surface. J Med Microbiol 56 77–82. 10.1099/jmm.0.46810-0 17172521

[R34] Xing Y. Yu T. Wu Y. N. Roy M. Kim J. Lee C.(2006). An expectation-maximization algorithm for probabilistic reconstructions of full-length isoforms from splice graphs. Nucleic Acids Res 34 3150–3160. 10.1093/nar/gkl396 16757580 PMC1475746

